# Dietary Vitamin Intake and Central and Peripheral Arterial Stiffness in Patients with Long COVID: The BioICOPER Study

**DOI:** 10.3390/nu18142336

**Published:** 2026-07-16

**Authors:** David Arjol, Silvia Arroyo-Romero, Elena Navarro-Matías, Alicia Navarro-Cáceres, Nuria Suárez-Moreno, Andrea Domínguez-Martín, Manuel A. Gómez-Marcos, Marta Gómez-Sánchez, Leticia Gómez-Sánchez, BioICOPER Investigators Group

**Affiliations:** 1 Unidad de Investigación de Atención Primaria de Salamanca (APISAL), Avd. Portugal, 37005 Salamanca, Spain; darjol@ibsal.es (D.A.); silvia_ar@usal.es (S.A.-R.); enavarro@saludcastillayleon.es (E.N.-M.); alicia.nav@usal.es (A.N.-C.); nuria.suarez@usal.es (N.S.-M.); andreadm@usal.es (A.D.-M.); 2 Instituto de Investigación Biomédica de Salamanca (IBSAL), Paseo de San Vicente, 37007 Salamanca, Spain; 3 Red de Investigación Sobre Cronicidad, Atención Primaria y Promoción de la Salud (RICAPPS), 37005 Salamanca, Spain; 4 Departamento de Medicina, Facultad de Medicina, Universidad de Salamanca, Calle Alfonso X El Sabio s/n, 37007 Salamanca, Spain; 5 Servicio de Salud de Castilla y León-SACYL, Gestión Regional de Salud, 37005 Salamanca, Spain; 6 Servicio de Hospitalización Domiciliaria, Hospital Universitario Marqués de Valdecilla, 39008 Santander, Spain; martagmzsnchz@gmail.com; 7 Servicio de Urgencias, Hospital Universitario de La Paz, P. de Castellana, 261, 28046 Madrid, Spain; leticiagmzsnchz@gmail.com

**Keywords:** Long COVID, central arterial stiffness, peripheral arterial stiffness, vascular health, vitamin intake, diet quality

## Abstract

**Background**: Long COVID (LC) has been associated with persistent inflammation and endothelial dysfunction. Dietary components, particularly vitamins, are involved in inflammation, oxidative stress, and vascular homeostasis, and may influence arterial stiffness in this population. However, the relationship between dietary vitamin intake and vascular stiffness parameters in patients with LC remains poorly understood. **Objective**: To analyze the association between dietary vitamin intake and central and peripheral arterial stiffness, evaluated by carotid–femoral pulse wave velocity (cfPWV) and brachial–ankle pulse wave velocity (baPWV), in patients with LC. **Methods**: We conducted a cross-sectional study that included 304 patients with LC. Dietary vitamin intake was assessed using a validated 7-day dietary record (EVIDENT tool). Vascular assessment included cfPWV and baPWV, measured using validated devices (SphygmoCor^®^ and VaSera^®^). Multiple linear regression analyses were performed to evaluate the associations between vitamin intake and vascular parameters. Model 1 was adjusted for age and sex, while Model 2 additionally included the number of metabolic syndrome components, the SF-36 general health domain, and adherence to the Mediterranean diet. Correction for multiple comparisons was performed using the Benjamini–Hochberg procedure across 36 vitamin–outcome–model contrasts. **Results**: The mean age was 52.78 ± 11.91 years, and 207 participants were women (68.1%); men were older than women (*p* = 0.004). In multiple regression analysis, inverse estimates for vitamin B1 intake with cfPWV were observed in both Model 1 (β = −0.568; 95% CI: −1.016 to −0.119; *p* = 0.014) and Model 2 (β = −0.491; 95% CI: −0.923 to −0.059; *p* = 0.027). Vitamin B6 intake showed a similar pattern with cfPWV in Model 1 (β = −0.312; 95% CI: −0.606 to −0.018; *p* = 0.039) and Model 2 (β = −0.302; 95% CI: −0.586 to −0.018; *p* = 0.038). Vitamin B2 showed inverse but non-significant associations with cfPWV. No significant associations were observed between vitamin intake and baPWV. After FDR correction, no vitamin-arterial stiffness association remained statistically significant; the FDR-adjusted q-value for the B1/B6-cfPWV estimates was 0.347. **Conclusions**: In this exploratory cross-sectional study, habitual food-based vitamin intake was not significantly associated with central or peripheral arterial stiffness after correction for multiple comparisons. Vitamins B1 and B6 showed inverse nominal estimates with cfPWV; however, these estimates did not survive FDR correction and were not reproduced for baPWV. Longitudinal studies incorporating biochemical micronutrient markers, medication and supplementation data, inflammatory and homocysteine-related biomarkers, and repeated vascular assessment are needed to clarify whether vitamin status is related to vascular health among individuals with persistent post-COVID-19 symptoms.

## 1. Introduction

Long COVID (LC) syndrome affects a significant percentage of individuals following SARS-CoV-2 infection and constitutes a complex, multisystemic disorder characterized by a wide variety of persistent symptoms [1,2]. SARS-CoV-2 exhibits a marked vascular tropism, which can promote persistent endothelial alterations and a chronic pro-inflammatory state [3,4], potentially associated with increased arterial stiffness [3,5]. Furthermore, it has been reported that women with LC may present poorer arterial elasticity [6]. Central arterial stiffness, evaluated via carotid–femoral pulse wave velocity (cfPWV), and peripheral arterial stiffness, evaluated by brachial–ankle pulse wave velocity (baPWV), are currently considered subclinical target organ damage biomarkers [7] and independent predictors of increased cardiovascular morbidity and mortality [8,9,10,11,12,13]. In this regard, recent studies have shown increased arterial stiffness in patients diagnosed with LC, even in subjects who experienced mild forms of acute infection, suggesting the presence of accelerated vascular aging that is potentially independent of the initial severity of the acute infection [5,14].

In subjects with LC, nutrition could act as a relevant modulator of vascular health. In particular, vitamins participate in the regulation of oxidative stress, inflammation, homocysteine metabolism, and calcium homeostasis—processes directly involved in arterial stiffness [15,16]. Different vitamin groups may influence vascular biology through partially distinct but interconnected mechanisms. B-complex vitamins contribute to energy metabolism, methylation reactions, and one-carbon metabolism, with folate, vitamin B6, and vitamin B12 being particularly involved in homocysteine regulation. Among them, water-soluble B-complex vitamins play a central role in homocysteine metabolism [17]. Hyperhomocysteineemia has been associated with endothelial dysfunction and increased arterial stiffness [18]. Thus, folate is known not only to reduce homocysteine levels but also to improve nitric oxide bioavailability by stabilizing the endothelial nitric oxide synthase (eNOS) enzyme, thereby contributing to vascular function [19]. Likewise, observational studies in the general population have shown inverse associations between folate intake and the progression of both central and peripheral arterial stiffness, suggesting a potential role of these vitamins in vascular aging [20]. Along these lines, the longitudinal EVA study has provided evidence regarding the inverse association between vitamin B9 intake and the increase in arterial stiffness over a five-year period [21]. On the other hand, vitamin C is a water-soluble antioxidant with a relevant role in neutralizing oxidative stress, a process that is increased in LC due to persistent inflammatory activation [22]. Moreover, a meta-analysis suggests that antioxidant vitamin supplementation can reduce arterial stiffness, reinforcing its potential role in modulating vascular function [23]. Beyond antioxidant defense, vitamin C may also contribute to vascular homeostasis through pathways related to endothelial function and nitric oxide bioavailability. Among fat-soluble vitamins, the interaction between vitamin D and vitamin K has acquired special relevance in the regulation of calcium metabolism and the prevention of vascular calcification. Vitamin D is also involved in immune regulation and inflammatory signaling, mechanisms that may be particularly relevant in LC given the persistence of immune activation described in this condition. Recent studies have suggested that combined supplementation with vitamins D3 and K2 could improve inflammatory and oxidative stress markers in patients with LC, although evidence on arterial stiffness outcomes remains limited [24,25]. The role of other fat-soluble vitamins, such as vitamin A, remains more controversial. Although it exhibits antioxidant properties, clinical study results are inconsistent, showing variable effects depending on the dose and clinical context [26].

Furthermore, arterial stiffness is not homogeneous throughout the vascular tree. Central arteries, such as the aorta, depend to a greater extent on the structural integrity of elastin and the degree of calcification, whereas peripheral arteries are more influenced by vasomotor tone mediated by endothelial function. This heterogeneity suggests that the impact of vitamin intake could differ between central and peripheral arterial stiffness, which justifies the joint evaluation of cfPWV and baPWV [27,28].

Despite advances in this field, a relevant knowledge gap persists regarding the relationship between overall dietary vitamin intake and arterial stiffness in patients with Long COVID. Most previous studies have focused on individual vitamins or supplementation interventions, without considering habitual dietary intake across multiple vitamins, the co-occurrence of micronutrients within dietary patterns, or their potential differential association with distinct vascular territories. This distinction is relevant because vitamins do not act as isolated exposures in usual diets. Their biological effects are embedded within food matrices, correlated nutrient intakes, and interacting metabolic networks; therefore, vitamin-specific estimates may partly reflect broader diet quality or micronutrient clustering rather than the isolated effect of a single vitamin. In this context, the use of previously validated dietary tools, such as EVIDENT, allows for a systematic estimation of habitual vitamin intake [29].

Based on the above, the present study aimed to analyze the relationship between dietary vitamin intake and central and peripheral arterial stiffness, evaluated by cfPWV and baPWV, in patients with LC. Given the involvement of vitamins in antioxidant defense, homocysteine metabolism, calcium homeostasis, and immune-inflammatory regulation, we hypothesized that higher vitamin intake could be associated with lower arterial stiffness values in this population.

## 2. Materials and Methods

### 2.1. Study Design and Participants

This cross-sectional study was carried out in 304 subjects diagnosed with LC. The study was conducted at the Primary Care Research Unit of Salamanca (APISAL). This manuscript is part of the BioICOPER project, which was registered in April 2023 at ClinicalTrials.gov (Identifier: NCT05819840). The study protocol has been previously published [30].

**Figure 1:**
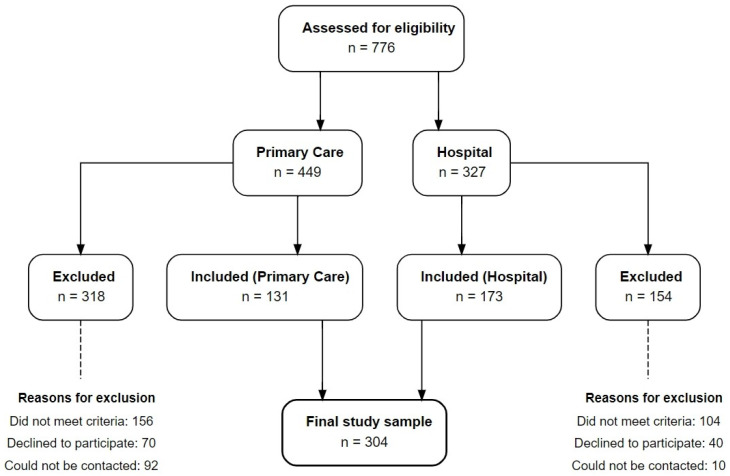
Flowchart of participant selection in the BioICOPER study according to recruitment source. Participants were initially assessed from primary care and hospital pathways and subsequently included in the final analytical sample. Exclusion reasons are shown for each recruitment source.

Subjects were included using consecutive sampling. The diagnosis of LC was established according to the World Health Organization (WHO) criteria [31], and all participants provided written informed consent prior to their inclusion in the study. Exclusion criteria were having a terminal illness, being unable to attend the primary care center for evaluation, having a history of cardiovascular disease, or presenting an estimated glomerular filtration rate below 30 mL/min/1.73 m^2^. The participant selection process is shown in Figure 1.

The sample size and the detectable effect size were estimated using the GRANMO software v.5 (https://www.datarus.eu/ca/aplications/granmo/, accessed on 23 April 2026). For a multiple linear regression analysis with a total sample of 304 participants, five covariates, an alpha risk of 0.05, a statistical power greater than 80%, and an expected *R*^2^ close to that observed in the main models, the study allowed for the detection of an approximate minimum partial correlation of 0.16 between a continuous nutritional exposure and cfPWV.

The study followed the STROBE statement recommendations for observational studies [32]. The STROBE checklist is provided as Supplementary Checklist Table S1.

### 2.2. Variables and Measurement Instruments

To reduce information bias, four healthcare professionals were previously trained following a standardized protocol to perform all study assessments. Variables were recorded using REDCap (Research Electronic Data Capture), hosted at the Biomedical Research Institute of Salamanca (IBSAL), for data collection and management. Quality control was performed by an independent investigator [30].

### 2.3. Dietary Intake Assessment

Vitamin intake was assessed using dietary records collected through the EVIDENT mobile application [33]. This application was created and validated by our Primary Care Research Group of Castilla y León (REDIAPP) and is registered under intellectual property number 00/2014/2207. Participants in the BioICOPER study recorded all food and beverages consumed over seven consecutive days, including portion sizes and cooking methods. Foods were classified into predefined groups within the EVIDENT tool. Daily vitamin intake was estimated using the Spanish nutrient-composition data implemented in EVIDENT, including vitamin A, B-complex vitamins, vitamin C, and vitamin D, expressed in the corresponding units for each micronutrient [33]. These estimates reflected food-based intake from the 7-day dietary record.

The adequacy of vitamin intake was evaluated by comparing the estimated mean daily intake with dietary reference values for the adult population, applying sex-specific cut-off points when applicable [34,35,36]. Adequate intake was defined as achieving at least 750 µg/day of retinol equivalents for vitamin A in men and 650 µg/day in women; 1.2 and 0.9 mg/day for thiamine; 1.6 and 1.3 mg/day for riboflavin; 16 and 14 mg/day for niacin equivalents; 1.7 and 1.6 mg/day for vitamin B6; and 110 and 95 mg/day for vitamin C, respectively. For recommendations common to both sexes, the cut-off points were 330 µg/day of dietary folate equivalents for vitamin B9, 4.0 µg/day for vitamin B12, and 15 µg/day for vitamin D. Based on these cut-off points, participants were classified as compliers or non-compliers with the corresponding recommendation for each micronutrient.

### 2.4. Assessment of Vascular Structure, Function, and Aging

All measurements were performed under baseline conditions, after 10 min of rest in the supine position, in a temperature-controlled room.

#### Arterial Stiffness Measurements

Arterial stiffness was assessed by measuring carotid–femoral pulse wave velocity (cfPWV) and brachial–ankle pulse wave velocity (baPWV).

Central arterial stiffness was evaluated using carotid–femoral pulse wave velocity (cfPWV) measured with the SphygmoCor system (AtCor Medical Pty Ltd., West Ryde, Australia) with the participant in the supine position. Pulse waves were recorded at the carotid and femoral arteries. Transit time was estimated relative to the R-wave of the electrocardiogram. Distances were measured with a tape measure from the suprasternal notch to the carotid and femoral recording sites. cfPWV was calculated according to established measurement guidelines [7,37,38].

baPWV was measured using the VaSera VS-2000 device (Fukuda Denshi Co., Ltd., Tokyo, Japan). Electrodes were placed on both arms and ankles, and measurements were performed with the participant in the supine position, remaining silent and motionless.

According to the manufacturer’s instructions, baPWV was calculated using the following equation:

baPWV=(0.5934∗height(cm)+14.4724)/tba

baPWV was derived from the ratio between the estimated length—taking into account each subject’s height—and the pulse transit time between the brachial and ankle sites, reflecting arterial stiffness [8,39,40].

### 2.5. Sociodemographic Variables, Lifestyle Factors, and Laboratory Analyses

Sociodemographic, clinical, and lifestyle variables were collected using standardized questionnaires. Age and sex were recorded, alongside tobacco and alcohol consumption, which were evaluated using questionnaires adapted from the WHO MONICA study [41]. Adherence to the Mediterranean diet was assessed using the 14-item Mediterranean Diet Adherence Screener (MEDAS), the score of which was used as an overall indicator of dietary pattern quality [42]. Physical activity was measured using a validated digital pedometer (Omron HJ-321-E, Omron Healthcare Co., Ltd., Kyoto, Japan), which recorded total steps, aerobic steps, distance traveled (km), and calories expended during the previous seven days [43]. Health-related quality of life was evaluated using the Short Form-36 Health Survey (SF-36) questionnaire, employing the general health domain as an indicator of current perceived health status [44]. Fasting blood samples were obtained to determine plasma glucose, total cholesterol, low-density lipoprotein cholesterol, high-density lipoprotein cholesterol, and triglycerides. Blood pressure and heart rate were measured using a validated automated sphygmomanometer (OMRON M10-IT, Omron Healthcare Co., Ltd., Kyoto, Japan), following the European Society of Hypertension recommendations [7]. Cardiometabolic burden was summarized by the number of metabolic syndrome components, considering elevated blood pressure, elevated triglycerides, low HDL cholesterol, elevated blood glucose, and abdominal obesity, following the international consensus recommendations of the National Cholesterol Education Program Expert Panel on Detection, Evaluation, and Treatment of High Blood Cholesterol in Adults (Adult Treatment Panel III) [45]. Vaccination-related information was recorded, including the number of COVID-19 vaccine doses, vaccine type, and vaccination dates. SARS-CoV-2-related serological measurements included N antigen and anti-S IgG levels determined by ELISA.

### 2.6. Statistical Analysis

The normality of continuous variables was examined using the Shapiro–Wilk test, skewness, kurtosis, and graphical inspection of histograms, boxplots, and quantile–quantile plots. Continuous variables were described as mean ± standard deviation or median and interquartile range, depending on their distribution, and categorical variables as n (%). Comparisons by sex were performed using Student’s *t*-test, with Welch’s correction when necessary, or the Mann–Whitney test for continuous variables, and the chi-squared test or Fisher’s exact test for categorical variables. Missing data were examined by variable and by analytical model. Multivariable models were estimated using complete-case analysis. To assess potential selection related to missingness, participants included in the primary complete-case model were compared with those excluded from that model.

Nutritional adequacy was described for descriptive purposes only as the proportion of participants meeting the dietary recommendations for each vitamin. Associations between vitamin intake and central and peripheral arterial stiffness were estimated using separate multiple linear regression models for cfPWV and baPWV, with each vitamin entered as a continuous exposure. Models using continuous vitamin intake were considered the primary analyses.

Two prespecified models were adjusted based on clinical and epidemiological criteria defined a priori. Model 1 included age and sex. Model 2 additionally included the number of metabolic syndrome components, the SF-36 general health domain, and adherence to the Mediterranean diet, and was considered the main adjusted model. Sex-specific effect modification was assessed by adding vitamin × sex interaction terms to Model 2; these analyses were considered exploratory. Additional exploratory effect-modification analyses were performed for metabolic syndrome burden and Mediterranean diet adherence.

Model assumptions were evaluated by inspecting residuals, fitted values, and quantile–quantile plots. Multicollinearity and influential observations were assessed using the variance inflation factor (VIF), leverage, Cook’s distance, and studentized residuals. HC3 robust standard errors were used for all vitamin–arterial stiffness regression models.

Correction for multiple comparisons was performed using the Benjamini–Hochberg procedure to control the false discovery rate across the prespecified vitamin–outcome contrast family. This family comprised nine vitamins, two arterial stiffness outcomes, and two adjustment models, yielding 36 contrasts. Additional sensitivity summaries examined FDR correction within restricted contrast families, including Model 2 contrasts only and cfPWV Model 2 contrasts only.

Exploratory robustness analyses were conducted to assess the stability of the vitamin–arterial stiffness estimates. These analyses included staged adjustment models; separate models additionally adjusted for mean, systolic, and diastolic blood pressure; adiposity-related adjustment when available; influence diagnostics excluding high-influence or high-leverage observations; and a standardized B-complex index. Additional sensitivity models for the B1/B6–cfPWV and B1/B6–baPWV estimates incorporated available SARS-CoV-2- and vaccination-related variables, including number of COVID-19 vaccine doses, SARS-CoV-2 N antigen, and anti-S IgG levels. A further sensitivity analysis was performed after excluding participants who reported dietary supplement use. Sensitivity models were interpreted separately from the primary FDR-corrected vitamin–outcome contrast family. Potential reverse causation or health-conscious behavior was explored by testing whether vascular or cardiometabolic risk markers were associated with vitamin intake or Mediterranean diet adherence. All analyses were conducted using R version 4.4.2 (R Foundation for Statistical Computing, Vienna, Austria). Model diagnostic evaluation was carried out using the *car* package, and robust standard errors were computed using *lmtest* and *sandwich*. Statistical significance for the vitamin–arterial stiffness association analyses was determined using FDR-adjusted q-values, with q < 0.05 considered statistically significant.

### 2.7. Ethical Considerations

This study was approved on 27 June 2022 (CEIm: Ref. PI 2022 06 1048) by the “Ethics Committee for Research with Medicines of the Salamanca Health Area”. Throughout the study, the standards of good practice in observational studies established by the Declaration of Helsinki and by the WHO were followed [46]. The confidentiality of the participants was guaranteed at all times in accordance with Organic Law 3/2018, European Regulation 2016/679, and Council Directive 27 April 2016 on Data Protection. All analyzed subjects provided signed informed consent prior to inclusion in the study and after receiving detailed information about the procedures to be performed.

### 2.8. Use of Artificial Intelligence Tools

During the preparation of this manuscript, ChatGPT (OpenAI, GPT-5.5 Thinking) was used to assist with English language editing and with the formatting and assembly of manuscript figures based on the author-generated results. Generative AI was not used to create scientific images, generate data, perform analyses, or alter study findings. All outputs were checked against the original data and statistical results by the authors. The authors reviewed and approved all AI-assisted edits and assume full responsibility for the final manuscript.

## 3. Results

### Participant Characteristics at Study Inclusion

The sample comprised 304 participants, of whom 207 were women (68.1%). Compared with women, men were older (*p* = 0.004), had higher systolic, diastolic, and mean blood pressure values, and showed a higher prevalence of hypertension, diabetes mellitus, and obesity (all *p* < 0.001). Likewise, men had higher cfPWV and baPWV values (both *p* ≤ 0.001). The remaining sociodemographic, clinical, vascular, and vaccination-related characteristics of the sample are presented in Table 1.

**Figure 2:**
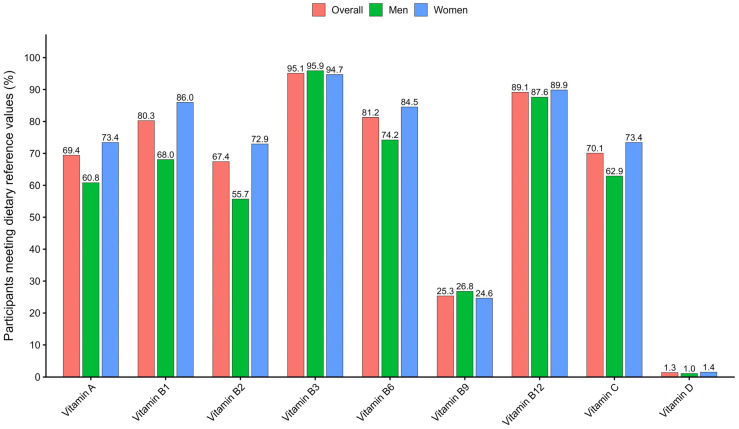
Proportion of participants meeting dietary reference values for each vitamin, overall and by sex. Percentages represent dietary intake from recorded foods and beverages only; vitamin intake from dietary supplements was not included in these estimates. Adequacy variables were used for descriptive purposes only.

No between-sex differences were observed in mean intake for any of the analyzed vitamins (all *p* > 0.05). However, women had a higher proportion of participants meeting dietary recommendations for vitamin A (*p* = 0.014), vitamin B1 (*p* < 0.001), vitamin B2 (*p* = 0.001), and vitamin B6 (*p* = 0.011). Vitamin intake values are shown in Table 2, and the proportion of participants meeting dietary recommendations is shown in Figure 2.

In the complete-case regression models, vitamins B1 and B6 showed inverse nominal estimates with cfPWV, but these estimates did not remain statistically significant after correction for multiple comparisons. The comparison between participants included and excluded from the main complete-case analysis is presented in Supplementary Table S2. For cfPWV, the sample size was 289 participants in Model 1 and 283 in Model 2. Vitamin B1 showed an inverse estimate with cfPWV in Model 1 (β = −0.568; robust SE = 0.229; 95% CI: −1.016 to −0.119; *p* = 0.014) and Model 2 (β = −0.491; robust SE = 0.220; 95% CI: −0.923 to −0.059; *p* = 0.027). Vitamin B6 showed a similar pattern in Model 1 (β = −0.312; robust SE = 0.150; 95% CI: −0.606 to −0.018; *p* = 0.039) and Model 2 (β = −0.302; robust SE = 0.145; 95% CI: −0.586 to −0.018; *p* = 0.038). After Benjamini–Hochberg correction across the 36 vitamin–outcome–model contrasts, none of these associations remained statistically significant. The FDR-adjusted q-value for the B1/B6-cfPWV estimates was 0.347. No other vitamin showed evidence of a statistically significant association with cfPWV after FDR correction.

In the baPWV models, estimated with 287 participants in Model 1 and 281 in Model 2, no vitamin showed evidence of a statistically significant association after FDR correction. For vitamin B1, the estimates were β = −0.284 in Model 1 (95% CI: −0.656 to 0.087; *p* = 0.135) and β = −0.170 in Model 2 (95% CI: −0.519 to 0.180; *p* = 0.342). For vitamin B6, the corresponding estimates were β = −0.138 (95% CI: −0.376 to 0.100; *p* = 0.257) and β = −0.116 (95% CI: −0.340 to 0.107; *p* = 0.309). The complete regression results for cfPWV and baPWV are presented in Table 3 FDR-adjusted q-values are provided in Supplementary Table S3, and the model diagnostic summary is available in Supplementary Table S4. Exploratory effect-modification analyses did not support differential B1/B6–cfPWV estimates by sex, metabolic syndrome burden, or Mediterranean diet adherence. The corresponding interaction models are reported in Supplementary Table S5. Vaccination-related information was available for all participants, and SARS-CoV-2 N antigen and anti-S IgG ELISA measurements were available for 294 participants. N antigen was equal to zero in 284 participants and detectable in 10 participants. Vaccination and SARS-CoV-2 serological data and the corresponding sensitivity models are reported in Supplementary Table S6. In these analyses, the B1–cfPWV estimate remained inverse after additional adjustment for vaccine doses and SARS-CoV-2 ELISA variables (β = −0.508; 95% CI: −0.975 to −0.042; *p* = 0.033; FDR q = 0.109), whereas the B6–cfPWV estimate was attenuated and was no longer nominally significant (β = −0.275; 95% CI: −0.577 to 0.027; *p* = 0.074; FDR q = 0.147). No corresponding B1/B6 pattern was observed for baPWV. After excluding the 11 participants who reported dietary supplement use, the B1–cfPWV and B6–cfPWV estimates in Model 2 remained nominally significant but not significant after FDR correction (B1: β = −0.497; 95% CI: −0.936 to −0.059; *p* = 0.026; FDR q = 0.280; B6: β = −0.321; 95% CI: −0.612 to −0.029; *p* = 0.031; FDR q = 0.280). In exploratory sensitivity analyses additionally adjusting Model 2 for blood pressure variables in separate models, the B1–cfPWV estimate remained stable after adjustment for mean blood pressure (β = −0.509; unadjusted *p* = 0.019), systolic blood pressure (β = −0.506; unadjusted *p* = 0.020), or diastolic blood pressure (β = −0.507; unadjusted *p* = 0.020). The B6–cfPWV estimate was slightly attenuated after adjustment for mean blood pressure (β = −0.277; unadjusted *p* = 0.052), systolic blood pressure (β = −0.280; unadjusted *p* = 0.053), or diastolic blood pressure (β = −0.280; unadjusted *p* = 0.050).

## 4. Discussion

This study provides new insights into the relationship between food-based vitamin intake and vascular health in patients with LC. To the best of our knowledge, this is one of the first studies to integrate a detailed assessment of dietary vitamin intake with an evaluation of both central and peripheral arterial stiffness in this specific population. Overall, habitual food-based vitamin intake was not robustly associated with central or peripheral arterial stiffness after correction for multiple comparisons. Vitamins B1 and B6 showed inverse nominal estimates with cfPWV, but these estimates did not survive FDR correction and were not reproduced for baPWV. This pattern is consistent with the current evidence base: vascular dysfunction and increased arterial stiffness have been described in LC, whereas direct evidence linking individual dietary vitamin intake to cfPWV or baPWV in this population remains scarce [3,5,14,15,26].

Regarding vitamin B1, the inverse cfPWV estimate is biologically plausible, although the available evidence does not support a confirmatory interpretation. Most prior studies have focused chiefly on vitamins B6, B9, and B12, while specific evidence concerning B1 and arterial stiffness remains limited [15,26]. Thiamine participates in mitochondrial energy metabolism and may be relevant to oxidative stress and endothelial function, mechanisms also implicated in LC [3,4,26]. In this context, the observed B1-cfPWV pattern may reflect residual confounding, broader dietary patterns, or chance variation rather than a vitamin-specific vascular effect. The data do not establish a differential vascular relevance of vitamin B1 in LC.

Regarding vitamin B6, the observed estimate is also biologically plausible but should be interpreted within the same exploratory framework. Vitamin B6 participates in the homocysteine transsulfuration pathway, which may contribute to modulating oxidative stress and vascular dysfunction [17,18,19]. Observational studies and meta-analyses have shown that elevated homocysteine levels are associated with higher cfPWV and accelerated vascular aging [17,19]. However, evidence linking homocysteine-related pathways to arterial stiffness does not imply that dietary B6 intake has an independent protective effect in LC. The present findings should therefore be viewed as preliminary and require confirmation using longitudinal designs and biochemical markers.

Concerning vitamin B2, we observed an inverse trend with cfPWV that did not reach statistical significance. This pattern is compatible with, but does not confirm, previous population-based evidence suggesting vascular associations for riboflavin and folate [16,21]. In observational dietary analyses, isolated vitamin-specific interpretations are intrinsically difficult because B-vitamin intakes tend to cluster within broader dietary patterns. Thus, the B-vitamin estimates observed here may partly reflect overall diet composition rather than specific effects of individual micronutrients [16,21,26]. More broadly, these findings should be interpreted within the context of correlated micronutrient intake and dietary pattern structure. Vitamins participate in overlapping metabolic networks rather than acting as isolated exposures, and this limits the attribution of vascular estimates to single micronutrients in observational dietary studies.

In this study, we found no associations with vitamin B9. This null finding is noteworthy because folate is one of the dietary vitamins with comparatively more direct longitudinal evidence in relation to arterial stiffness progression. The EVA Follow-Up 3 Study reported that lower dietary intakes of vitamin B9 and vitamin C were associated with greater progression of both cfPWV and baPWV in adults without previous cardiovascular disease [21]. This result is also compatible with a meta-analysis concluding that folic acid might have favorable effects on endothelial function, although effects on arterial stiffness markers appear less consistent [47]. The discrepancy with our findings may reflect differences in population characteristics, baseline nutritional status, vascular phenotype, exposure measurement, or the specific inflammatory and immunometabolic context of LC. Dietary intake alone may also be insufficient to capture folate-related biological pathways, particularly in the absence of serum folate, vitamin B12, methylmalonic acid, and homocysteine measurements.

Similarly, we observed no significant associations between vitamin C and arterial stiffness. This result contrasts with longitudinal evidence in which vitamin C, together with folate, has been related to cfPWV and baPWV progression [21], and with previous work suggesting that antioxidant vitamin supplementation may reduce PWV [22,23]. However, several factors could explain this discrepancy. First, much of the prior evidence comes from pharmacological supplementation studies, longitudinal general-population cohorts, or populations under different oxidative stress conditions. Second, in our cohort, the mean intake of vitamin C was relatively high, with more than 70% of participants meeting dietary recommendations, which may have reduced the variability necessary to detect significant associations. Furthermore, vitamin C intake may be insufficient on its own to represent antioxidant capacity in the complex inflammatory and endothelial environment of LC. Therefore, the absence of an association does not exclude a role for antioxidant status, but it indicates that habitual dietary vitamin C intake was not clearly related to cfPWV or baPWV in this sample.

Regarding the fat-soluble vitamins A and D, no significant associations were observed with either cfPWV or baPWV. In the case of vitamin D, these findings must be interpreted with caution, given that the biological status of this vitamin depends not only on dietary intake but also on sun exposure, cutaneous synthesis, seasonality, adiposity, supplement dose and duration, and other metabolic factors that were not quantified in this study [15,26]. Moreover, although recent studies suggest a potential biological relevance of combined vitamin D3-K2 supplementation in patients with LC [24,25], this evidence mainly concerns symptoms, inflammatory markers, oxidative stress-related parameters, and related biological pathways rather than cfPWV or baPWV outcomes. Thus, those findings cannot be directly compared with the dietary vitamin D analyses reported here.

Finally, the absence of significant associations with baPWV suggests that no consistent vitamin-related pattern was observed for mixed central-peripheral arterial stiffness. The B1/B6 estimates were restricted to cfPWV, which may be relevant because cfPWV more directly reflects aortic stiffness, whereas baPWV incorporates both central and peripheral arterial segments [27,28]. Additional adjustment for mean, systolic, or diastolic blood pressure in separate models left the B1-cfPWV estimate essentially unchanged and slightly attenuated the B6-cfPWV estimate, suggesting that blood pressure-related pathways may partly contribute to the observed B6 pattern. Nevertheless, the present results do not establish a selective effect of vitamin intake on central arterial stiffness. They indicate, rather, that any potential dietary vitamin signal was weak and limited to cfPWV.

From a clinical perspective, these findings do not support vitamin-specific supplementation strategies aimed at reducing arterial stiffness in patients with LC. Nutritional assessment may still be appropriate within comprehensive care, particularly to identify suboptimal dietary patterns or clinically relevant micronutrient inadequacies according to standard nutritional criteria. However, the present data do not provide evidence that food-based vitamin intake is independently or robustly associated with central or peripheral arterial stiffness.

### Strengths and Limitations

This study presents several limitations that should be considered when interpreting the results. First, its observational design precludes establishing causal relationships or completely ruling out the effect of unmeasured confounding variables. Second, vitamin intake was assessed using a 7-day dietary record; although a validated tool was employed, it is subject to self-report bias and may not accurately reflect long-term habitual intake. Third, dietary intake does not necessarily reflect circulating micronutrient status, which may be influenced by absorption, metabolism, inflammation, medication use, supplementation, and other non-dietary determinants. Although dietary supplement use was recorded, information on supplement type, dose, frequency, duration, and micronutrient composition was unavailable; therefore, supplement-derived vitamin intake could not be incorporated into the dietary intake estimates. Fourth, although vaccination-related information and SARS-CoV-2 N antigen and anti-S IgG ELISA measurements were available and examined in exploratory sensitivity analyses, these variables were not part of the prespecified primary analytical framework. Fifth, some variables included in the adjusted models, such as blood pressure-related and cardiometabolic factors, could partly lie on the pathway between vitamin intake and arterial stiffness; therefore, the adjusted estimates should be interpreted as conservative associations rather than estimates of the total potential dietary effect. Sixth, although complete-case comparisons were performed to explore potential selection related to missingness, some influence of missing data on the estimates cannot be fully excluded. Finally, although the sample size allowed the detection of moderate associations, it may have been insufficient to identify small effects after multivariable adjustment and correction for multiple vitamin–outcome comparisons; therefore, a type II error cannot be excluded.

The study also has several strengths. To the best of our knowledge, it is one of the first studies to examine habitual dietary vitamin intake in relation to both central and peripheral arterial stiffness in patients with LC. Vitamin intake was estimated using a validated dietary assessment tool, and arterial stiffness was assessed using two complementary vascular phenotypes: cfPWV, reflecting aortic stiffness, and baPWV, reflecting mixed central–peripheral arterial stiffness. The analysis also incorporated prespecified multivariable models, robust HC3 standard errors, FDR correction across a predefined contrast family, complete-case comparisons, model diagnostics, exploratory sensitivity analyses incorporating available vaccination-related variables and SARS-CoV-2 serological markers, sensitivity analyses excluding participants who reported dietary supplement use, and exploratory effect-modification analyses.

## 5. Conclusions

In this exploratory cross-sectional study of patients with LC, habitual food-based vitamin intake was not significantly associated with central or peripheral arterial stiffness after correction for multiple comparisons. Vitamins B1 and B6 showed inverse nominal estimates with cfPWV; however, these estimates did not survive FDR correction and were not reproduced for baPWV. Future longitudinal studies incorporating biochemical micronutrient markers, medication and supplementation data, inflammatory and homocysteine-related biomarkers, and repeated vascular assessment are needed to clarify whether vitamin status is related to vascular health among individuals with persistent post-COVID-19 symptoms.

## Figures and Tables

**Table 1:** Characteristics of the study population overall and by sex.

Variable	Reference Value or Clinical Cut-Off	Overall (*n* = 304)	Men (*n* = 97)	Women (*n* = 207)	*p*-Value
Age, years	—	52.78 ± 11.91	55.70 ± 12.28	51.41 ± 11.51	0.004
Time of evolution, months	—	40.23 (34.85–46.17)	40.50 (33.20–46.57)	40.13 (35.63–45.10)	0.991
Alcohol, g/week	—	0.00 (0.00–30.00)	25.00 (0.00–95.00)	0.00 (0.00–20.00)	<0.001
No alcohol consumption, n (%)	—	163 (53.6)	36 (37.1)	127 (61.4)	<0.001
MD score	Higher score = greater adherence	8.00 (7.00–9.00)	8.00 (6.00–9.00)	8.00 (7.00–9.00)	0.424
MD adherence, n (%)	MEDAS ≥ 9	123 (40.5)	38 (39.2)	85 (41.1)	0.755
MET-min/week	—	3360 (1680–7320)	3480 (1800–6480)	2880 (1560–8160)	0.246
Number of steps per day	—	6869 (4753–9251)	6041 (4756–8639)	7344 (4753–9503)	0.161
Years of smoking	—	22.00 (14.00–31.00)	27.50 (16.00–34.00)	19.00 (14.00–29.00)	0.074
Current smoker, n (%)	Current smoking	16 (5.3)	8 (8.2)	8 (3.9)	0.111
No. of cigarettes/day	**—**	14.50 (8.00–20.00)	20.00 (10.00–25.00)	10.00 (8.00–20.00)	0.001
SBP, mmHg	<140 mmHg	118.00 (108.00–131.25)	128.50 (119.50–139.00)	112.50 (104.00–126.50)	<0.001
DBP, mmHg	<90 mmHg	76.88 ± 11.12	82.34 ± 11.04	74.33 ± 10.22	<0.001
MBP, mmHg	**—**	91.25 ± 12.43	98.04 ± 11.33	88.06 ± 11.64	<0.001
Hypertension, n (%)	≥140/90 mmHg, diagnosis, or treatment	109 (35.9)	52 (53.6)	57 (27.5)	<0.001
Total cholesterol, mg/dL	<200 mg/dL	186 (163–207)	182 (159–200)	188 (166–211)	0.073
LDL-c, mg/dL	<100 mg/dL	111 (92–132)	114 (91–131)	110 (93–132)	0.724
HDL-c, mg/dL	≥40 men; ≥50 women	56 (46–66)	46 (40–56)	61 (51–68)	<0.001
Triglycerides, mg/dL	<150 mg/dL	87 (67–121)	104 (82–141)	82 (64–108)	<0.001
Dyslipidemia, n (%)	Diagnosis, treatment, or abnormal lipid profile	201 (66.1)	71 (73.2)	130 (62.8)	0.074
FPG, mg/dL	<100 mg/dL	84 (79–91)	88 (83–100)	82 (78–88)	<0.001
Diabetes mellitus, n (%)	FPG ≥ 126 mg/dL, diagnosis, or treatment	37 (12.2)	22 (22.7)	15 (7.2)	<0.001
Weight, kg	—	74.65 (61.35–87.55)	87.30 (78.00–94.90)	65.60 (58.60–79.30)	<0.001
Height, cm	—	163.75 (159.00–170.00)	172.50 (167.50–177.00)	161.00 (157.00–165.00)	<0.001
BMI, kg/m^2^	<30 kg/m^2^	27.60 (23.60–31.20)	29.40 (26.00–32.50)	26.40 (22.60–30.60)	<0.001
Obesity, n (%)	BMI ≥ 30 kg/m^2^	99 (32.6)	44 (45.4)	55 (26.6)	0.001
cfPWV, m/s	<10 m/s	7.40 (6.20–8.90)	8.00 (6.90–9.90)	7.00 (6.00–8.10)	<0.001
baPWV, m/s	No universal cut-off	12.31 (11.09–14.04)	13.29 (12.09–15.11)	11.96 (10.91–13.47)	<0.001
Vaccine doses (mean)		2.93 ± 1.17	3.11 ± 1.24	2.85 ± 1.13	0.072

Values are presented as mean ± standard deviation or median (interquartile range) for continuous variables and as n (%) for categorical variables. Reference values or clinical cut-off points are provided only to facilitate descriptive interpretation and were not used to define the primary regression models or participant eligibility. Dashes indicate variables for which no single clinical cut-off was applied in this study. For cfPWV, values ≥10 m/s are commonly interpreted as increased central arterial stiffness. For baPWV, proposed thresholds vary across populations and clinical contexts; therefore, no universal cut-off was applied. Dyslipidemia was defined as previous clinical diagnosis, lipid-lowering treatment, or abnormal lipid profile at assessment. Between-sex comparisons were performed using Student’s *t*-test with Welch correction when appropriate, the Mann–Whitney U test, the chi-squared test, or Fisher’s exact test, as appropriate. Abbreviations: baPWV, brachial–ankle pulse wave velocity; BMI, body mass index; cfPWV, carotid–femoral pulse wave velocity; DBP, diastolic blood pressure; FPG, fasting plasma glucose; HDL-c, high-density lipoprotein cholesterol; LDL-c, low-density lipoprotein cholesterol; MBP, mean blood pressure; MD, Mediterranean diet; MEDAS, Mediterranean Diet Adherence Screener; MET, metabolic equivalent; SBP, systolic blood pressure.

**Table 2:** Vitamin intake according to dietary reference values, overall and by sex.

Variable	Overall (*n* = 304)	Men (*n* = 97)	Women (*n* = 207)	*p*-Value
Vitamin A, µg RE/day	982.34 ± 832.44	1069.46 ± 1227.03	941.21 ± 556.54	0.338
Vitamin B1, mg/day	1.49 ± 0.56	1.48 ± 0.53	1.50 ± 0.58	0.840
Vitamin B2, mg/day	1.69 ± 0.59	1.67 ± 0.61	1.70 ± 0.58	0.706
Vitamin B3, mg/day	33.65 ± 9.10	34.65 ± 10.17	33.18 ± 8.54	0.230
Vitamin B6, mg/day	2.42 ± 0.87	2.34 ± 0.83	2.46 ± 0.89	0.285
Vitamin B9, µg/day	281.56 ± 97.81	290.66 ± 105.49	277.26 ± 93.93	0.298
Vitamin B12, µg/day	11.00 ± 11.39	10.06 ± 9.07	11.44 ± 12.34	0.283
Vitamin C, mg/day	148.99 ± 69.19	145.96 ± 72.15	150.42 ± 67.89	0.617
Vitamin D, µg/day	5.69 ± 3.20	5.45 ± 2.65	5.80 ± 3.43	0.331

Vitamin intake is presented as mean ± standard deviation. Dietary adequacy is shown separately in Figure 2 as the percentage of participants meeting the sex-specific or common dietary reference value for each vitamin. Between-sex comparisons were performed using Student’s *t*-test for continuous variables and the chi-squared test or Fisher’s exact test for categorical variables, as appropriate. Adequacy variables were used for descriptive purposes only, whereas regression models used vitamin intake as a continuous exposure. Vitamin intake estimates were derived from recorded foods and beverages and did not quantify vitamin intake from dietary supplements. Vitamin A was expressed as retinol equivalents (RE), including provitamin A carotenoid contributions, and should not be interpreted as preformed retinol intake alone.

**Table 3:** Multivariable linear regression models of continuous vitamin intake and central and peripheral arterial stiffness.

Model	Vitamin	cfPWV, β (95% CI); Unadjusted *p*	baPWV, β (95% CI); Unadjusted *p*
Model 1	Vitamin A, per 100 µg RE/day	−0.006 (−0.028 to 0.016); *p* = 0.601	0.004 (−0.053 to 0.061); *p* = 0.897
Model 1	Vitamin B1, mg/day	−0.568 (−1.016 to −0.119); *p* = 0.014	−0.284 (−0.656 to 0.087); *p* = 0.135
Model 1	Vitamin B2, mg/day	−0.374 (−0.751 to 0.003); *p* = 0.053	−0.200 (−0.548 to 0.148); *p* = 0.261
Model 1	Vitamin B3, mg/day	−0.019 (−0.046 to 0.008); *p* = 0.172	−0.010 (−0.034 to 0.014); *p* = 0.407
Model 1	Vitamin B6, mg/day	−0.312 (−0.606 to −0.018); *p* = 0.039	−0.138 (−0.376 to 0.100); *p* = 0.257
Model 1	Vitamin B9, µg/day	−0.001 (−0.003 to 0.002); *p* = 0.746	−0.001 (−0.003 to 0.002); *p* = 0.579
Model 1	Vitamin B12, µg/day	−0.008 (−0.028 to 0.011); *p* = 0.408	−0.002 (−0.028 to 0.024); *p* = 0.879
Model 1	Vitamin C, mg/day	−0.001 (−0.005 to 0.002); *p* = 0.468	−0.000 (−0.004 to 0.003); *p* = 0.846
Model 1	Vitamin D, µg/day	−0.038 (−0.101 to 0.025); *p* = 0.237	−0.006 (−0.069 to 0.058); *p* = 0.863
Model 2	Vitamin A, per 100 µg RE/day	−0.009 (−0.032 to 0.015); *p* = 0.462	0.001 (−0.052 to 0.053); *p* = 0.976
Model 2	Vitamin B1, mg/day	−0.491 (−0.923 to −0.059); *p* = 0.027	−0.170 (−0.519 to 0.180); *p* = 0.342
Model 2	Vitamin B2, mg/day	−0.331 (−0.690 to 0.028); *p* = 0.072	−0.142 (−0.465 to 0.182); *p* = 0.392
Model 2	Vitamin B3, mg/day	−0.019 (−0.046 to 0.009); *p* = 0.181	−0.009 (−0.030 to 0.013); *p* = 0.433
Model 2	Vitamin B6, mg/day	−0.302 (−0.586 to −0.018); *p* = 0.038	−0.116 (−0.340 to 0.107); *p* = 0.309
Model 2	Vitamin B9, µg/day	−0.001 (−0.003 to 0.002); *p* = 0.454	−0.001 (−0.003 to 0.001); *p* = 0.326
Model 2	Vitamin B12, µg/day	−0.008 (−0.025 to 0.009); *p* = 0.366	−0.001 (−0.025 to 0.023); *p* = 0.963
Model 2	Vitamin C, mg/day	−0.002 (−0.005 to 0.001); *p* = 0.257	−0.001 (−0.004 to 0.002); *p* = 0.561
Model 2	Vitamin D, µg/day	−0.028 (−0.089 to 0.033); *p* = 0.366	0.011 (−0.047 to 0.069); *p* = 0.716

Values are unstandardized regression coefficients with 95% confidence intervals and unadjusted p-values. Model 1 was adjusted for age and sex. Model 2 was additionally adjusted for the number of metabolic syndrome components, the SF-36 general health domain, and Mediterranean diet adherence. Each vitamin was entered as a continuous exposure in a separate model. For vitamin A, coefficients are expressed per 100 µg retinol equivalents/day to improve readability. Robust HC3 standard errors were used. After Benjamini–Hochberg correction across the 36 vitamin–outcome–model contrasts, no association remained statistically significant. The FDR-adjusted q-value for the B1/B6-cfPWV estimates was 0.347. Exploratory sensitivity analyses additionally adjusted for vaccination-related variables and SARS-CoV-2 serological markers are presented in Supplementary Table S6. Abbreviations: baPWV, brachial–ankle pulse wave velocity; cfPWV, carotid–femoral pulse wave velocity; CI, confidence interval; FDR, false discovery rate; RE, retinol equivalents.

## Data Availability

The data supporting the findings of this study are available on ZENODO at: https://doi.org/10.5281/zenodo.14282873.

## Supplementary Materials

The following supporting information can be downloaded at https://www.mdpi.com/article/10.3390/nu18142336/s1: Table S1: STROBE Statement checklist of items; Table S2: Comparison of participants with complete and incomplete data for the primary fully adjusted cfPWV model; Table S3: FDR-adjusted associations between vitamin intake and central and peripheral arterial stiffness in the primary models; Table S4: Diagnostic summary of the primary multivariable models; Table S5: Exploratory effect-modification analyses for the vitamin B1/B6-cfPWV estimates; Table S6: Exploratory adjustment of B1/B6–arterial stiffness models for vaccination and SARS-CoV-2 serological markers. Figure S1. Standardized associations between vitamin intake and central and peripheral arterial stiffness.

## Abbreviations

The following abbreviations are used in this manuscript:

LC	Long COVID
cfPWV	carotid–femoral pulse wave velocity
baPWV	brachial–ankle pulse wave velocity
APISAL	Salamanca Primary Care Research Unit
WHO	World Health Organization
STROBE	Strengthening the Reporting of Observational Studies in Epidemiology
REDCap	Research Electronic Data Capture
IBSAL	Biomedical Research Institute of Salamanca
SBP	systolic blood pressure
DBP	diastolic blood pressure
PWV	pulse wave velocity
FPG	Fasting plasma glucose
MEDAS	Mediterranean Diet Adherence Screener
HDL	High Density Lipoprotein
SF-36	Short Form Health Survey
BMI	Body mass index
VIF	Variance Inflation Factor
CI	Confidence interval
