# Supplementary material for: Dietary Vitamin Intake and Central and Peripheral Arterial Stiffness in Patients with Long COVID: The BioICOPER Study
Source: Nutrients. 2026 Jul 16;18(14):2336. doi: 10.3390/nu18142336 (PMC13415850; doi:10.3390/nu18142336)
Supplement: Supplementary file 1 [file nutrients-18-02336-s001.zip › nutrients-4423602-supplementary.pdf]

Table S1. STROBE Statement checklist of items

Item No.	STROBE recommendation	Page No.	Relevant text / section from manuscript
1(a)	Indicate the study's design with a commonly used term in the title or the abstract.	1	Abstract, Methods: the study is described as a cross-sectional analysis.
1(b)	Provide in the abstract an informative and balanced summary of what was done and what was found.	1	Abstract: structured summary including background, objective, methods, results, and conclusions, including the main FDR-adjusted interpretation of the vitamin–arterial stiffness analyses.
2	Explain the scientific background and rationale for the investigation being reported.	2	Section 1, Introduction: describes vascular alterations in Long COVID, persistent inflammatory and cardiometabolic mechanisms, and the potential relevance of dietary vitamin intake for vascular function.
3	State specific objectives, including any prespecified hypotheses.	3	Section 1, Introduction: the final paragraph states the objective of analyzing the association between dietary vitamin intake and central and peripheral arterial stiffness in patients with Long COVID.
4	Present key elements of study design early in the paper.	3	Section 2.1, Study design and participants: presents the cross-sectional design and the BioICOPER study setting.
5	Describe the setting, locations, and relevant dates, including periods of recruitment, exposure, follow-up, and data collection.	3	Section 2.1, Study design and participants: describes the Primary Care Research Unit of Salamanca setting and the BioICOPER project context.

Item No.	STROBE recommendation	Page No.	Relevant text / section from manuscript
6	Give the eligibility criteria, and the sources and methods of selection of participants.	3	Section 2.1, Study design and participants: describes the Long COVID definition, consecutive sampling, inclusion criteria, and exclusion criteria.
7	Clearly define all outcomes, exposures, predictors, potential confounders, and effect modifiers. Give diagnostic criteria, if applicable.	3-6	Sections 2.2, 2.3, and 2.4: define arterial stiffness outcomes, dietary vitamin intake exposures, cardiometabolic and clinical covariates, and exploratory effect modifiers.
8	For each variable of interest, give sources of data and details of methods of assessment.	3-6	Sections 2.2, 2.3, and 2.4: describe measurement protocols for dietary assessment, arterial stiffness measurements, clinical variables, anthropometry, blood pressure, and laboratory parameters.
9	Describe any efforts to address potential sources of bias.	3-4	Section 2.2, Variables and measurement instruments: describes standardized training of healthcare professionals and quality control procedures to reduce measurement and information bias.
10	Explain how the study size was arrived at.	4	Section 2.1, Study design and participants: describes the sample size rationale and statistical power calculation.
11	Explain how quantitative variables were handled in the analyses. If applicable, describe which groupings were chosen and why.	6	Section 2.6, Statistical analysis: vitamin intakes were modeled as continuous exposures in the primary regression analyses. Dietary adequacy categories were used for descriptive purposes only.
12(a)	Describe all statistical methods, including those used to control for confounding.	6	Section 2.6, Statistical analysis: separate multiple linear regression models were estimated for cfPWV and baPWV. Model 1 was adjusted for age and sex. Model 2

Item No.	STROBE recommendation	Page No.	Relevant text / section from manuscript
			was additionally adjusted for the number of metabolic syndrome components, SF-36 general health domain, and Mediterranean diet adherence.
12(b)	Describe any methods used to examine subgroups and interactions.	6	Section 2.6, Statistical analysis: exploratory effect-modification analyses were conducted by adding vitamin × sex interaction terms to Model 2. Additional exploratory interaction analyses evaluated metabolic syndrome burden and Mediterranean diet adherence as potential effect modifiers.
12(c)	Explain how missing data were addressed.	6	Section 2.6, Statistical analysis: missing data were examined by variable and analytical model; multivariable models were estimated using complete-case analysis.
12(d)	Cohort study: if applicable, explain how loss to follow-up was addressed. Case-control study: if applicable, explain how matching was addressed. Cross-sectional study: if applicable, describe analytical methods taking account of sampling strategy.	6	Section 2.6, Statistical analysis: analyses were based on the cross-sectional BioICOPER sample. Complete-case comparisons were used to assess potential selection related to missingness.
12(e)	Describe any sensitivity analyses.	6	Section 2.6, Statistical Analysis: sensitivity and robustness analyses included FDR correction within restricted contrast families, staged adjustment models, separate models additionally adjusted for mean, systolic, and diastolic blood pressure, adiposity-related adjustment when available, influence diagnostics, a standardized B-complex index, and additional exploratory models incorporating vaccination-related variables and SARS-CoV-2 serological markers.

Item No.	STROBE recommendation	Page No.	Relevant text / section from manuscript
13(a)	Report numbers of individuals at each stage of study.	3, 7	Section 2.1 and Section 3, Results: total sample size and analytical sample sizes for regression models are reported; participant selection is shown in Figure 1.
13(b)	Give reasons for non-participation at each stage.	3	Section 2.1 and Figure 1: participant flow and exclusions are described according to protocol criteria.
13(c)	Consider use of a flow diagram.	3	Section 2.1: the participant selection process is shown in Figure 1.
14(a)	Give characteristics of study participants and information on exposures and potential confounders.	7	Section 3, Results, and Tables 1 and 2: describe demographic, clinical, vascular, cardiometabolic, dietary, and vitamin intake characteristics.
14(b)	Indicate number of participants with missing data for each variable of interest.	6-7	Section 3, Results, and Supplementary Table S2: report complete-case and incomplete-case comparisons for the primary fully adjusted cfPWV model.
15	Cross-sectional study: report numbers of outcome events or summary measures.	7-9	Section 3, Results, and Tables 1-3: report summary measures for arterial stiffness outcomes, vitamin intake, dietary adequacy, and regression estimates.
16(a)	Give unadjusted estimates and, if applicable, confounder-adjusted estimates and their precision. Make clear which confounders were adjusted for and why they were included.	8-9	Section 3, Results, and Table 3: report Model 1 and Model 2 regression estimates with 95% confidence intervals and unadjusted p-values; Supplementary Table S3 reports FDR-adjusted q-values.
16(b)	Report category boundaries when continuous variables were categorized.	4-5	Section 2.3, Dietary assessment: describes dietary reference values used to describe nutritional adequacy. Adequacy categories were used for descriptive purposes only.

Item No.	STROBE recommendation	Page No.	Relevant text / section from manuscript
16(c)	If relevant, consider translating estimates of relative risk into absolute risk for a meaningful time period.	Not applicable	Not applicable; the study used cross-sectional linear regression models with continuous arterial stiffness outcomes.
17	Report other analyses done, such as analyses of subgroups and interactions, and sensitivity analyses.	7-9	Section 3, Results: reports primary regression results with FDR-adjusted q-values, model diagnostics, exploratory robustness analyses, and exploratory effect-modification analyses.
18	Summarize key results with reference to study objectives.	11	Section 4, Discussion: the opening paragraph summarizes the main findings in relation to the study objective and interprets the B1/B6-cfPWV estimates within an exploratory framework.
19	Discuss limitations of the study, taking into account sources of potential bias or imprecision. Discuss both direction and magnitude of any potential bias.	12	Section 4.1, Strengths and limitations: discusses the cross-sectional design, 7-day dietary assessment window, absence of biochemical vitamin markers, residual confounding, missing covariates such as medication use, sun exposure, socioeconomic status, and limited statistical power after multiplicity correction.
20	Give a cautious overall interpretation of results considering objectives, limitations, multiplicity of analyses, results from similar studies, and other relevant evidence.	11-13	Section 4, Discussion: provides a cautious interpretation considering the FDR-adjusted analyses, cross-sectional design, multiplicity correction, dietary assessment limitations, and the exploratory nature of the B1/B6-cfPWV estimates.
21	Discuss the generalisability of the study results.	11-13	Section 4 and Section 4.1: discusses generalisability within the context of Long COVID and the need for replication in larger, longitudinal, and biomarker-supported studies.

Item No.	STROBE recommendation	Page No.	Relevant text / section from manuscript
22	Give the source of funding and the role of the funders.	13	Funding section: identifies the funding sources and states that the funders had no role in study design, data analysis, interpretation of results, manuscript preparation, or the decision to submit the manuscript.

Table S2. Comparison of participants with complete and incomplete data for the primary fully adjusted cfPWV model.

Variable	Complete cases (*n* = 283)	Incomplete cases (*n* = 21)	*p*-value
Age, years	53.36 ± 11.75	44.86 ± 11.47	0.003
Female sex, n (%)	193 (68.2)	14 (66.7)	1.000
cfPWV, m/s	7.50 [6.20–9.10]	6.60 [6.15–7.35]	0.013
baPWV, m/s	12.39 [11.09–14.10]	12.02 [11.13–12.91]	0.116
Vitamin B1, mg/day	1.41 [1.14–1.77]	1.26 [1.22–1.37]	0.306
Vitamin B6, mg/day	2.31 [1.81–2.83]	2.22 [1.85–2.30]	0.401
Number of metabolic syndrome components	1.00 [0.00–2.00]	0.00 [0.00–2.00]	0.026
SF-36 general health domain	45.00 [30.00–62.00]	38.50 [25.00–57.00]	0.437
Mediterranean diet adherence	8.00 [7.00–9.00]	6.00 [0.00–9.00]	0.024

Complete cases were defined according to the variables required for the primary cfPWV Model 2, including cfPWV, vitamin intake variables, age, sex, number of metabolic syndrome components, the SF-36 general health domain, and Mediterranean diet adherence. Continuous variables are presented as mean ± standard deviation or median [interquartile range], according to their distribution. Comparisons were performed using Student's t-test or the Mann–Whitney U test for continuous variables and Fisher's exact test for categorical variables, as appropriate. Abbreviations: baPWV, brachial–ankle pulse wave velocity; cfPWV, carotid–femoral pulse wave velocity; SF-36, Short Form-36 Health Survey.

Table S3. FDR-adjusted associations between vitamin intake and central and peripheral arterial stiffness in the primary models.

Outcome	Model	Vitamin	β (robust SE, HC3)	95% CI	unadjusted *p*-value	FDR *q*-Value
cfPWV	Model 1	Vitamin A, per 100 µg RE/	-0.006 (0.011)	-0.028 to 0.016	.601	0.772
cfPWV	Model 1	day Vitamin B1, mg/day	-0.568 (0.229)	-1.016 to -0.119	.014	0.347
cfPWV	Model 1	Vitamin B2, mg/day	-0.374 (0.192)	-0.751 to 0.003	.053	0.379
cfPWV	Model 1	Vitamin B3, mg/day	-0.019 (0.014)	-0.046 to 0.008	.172	0.667
cfPWV	Model 1	Vitamin B6, mg/day	-0.312 (0.150)	-0.606 to -0.018	.039	0.347
cfPWV	Model 1	Vitamin B9, µg/day	-0.001 (0.001)	-0.003 to 0.002	.746	0.895
cfPWV	Model 1	Vitamin B12, µg/day	-0.008 (0.010)	-0.028 to 0.011	.408	0.667
cfPWV	Model 1	Vitamin C, mg/day	-0.001 (0.002)	-0.005 to 0.002	.468	0.674
cfPWV	Model 1	Vitamin D, µg/day	-0.038 (0.032)	-0.101 to 0.025	.237	0.667
cfPWV	Model 2	Vitamin A, per 100 µg RE/	-0.009 (0.012)	-0.032 to 0.015	.462	0.674
cfPWV	Model 2	day Vitamin B1, mg/day	-0.491 (0.220)	-0.923 to -0.059	.027	0.347
cfPWV	Model 2	Vitamin B2, mg/day	-0.331 (0.183)	-0.690 to 0.028	.072	0.431
cfPWV	Model 2	Vitamin B3, mg/day	-0.019 (0.014)	-0.046 to 0.009	.181	0.667
cfPWV	Model 2	Vitamin B6, mg/day	-0.302 (0.145)	-0.586 to -0.018	.038	0.347
cfPWV	Model 2	Vitamin B9, µg/day	-0.001 (0.001)	-0.003 to 0.002	.454	0.674

Outcome	Model	Vitamin	β (robust SE, HC3)	95% CI	unadjusted *p*-value	FDR *q*-Value
cfPWV	Model 2	Vitamin B12, μg/day	-0.008 (0.009)	-0.025 to 0.009	.366	0.667
cfPWV	Model 2	Vitamin C, mg/day	-0.002 (0.002)	-0.005 to 0.001	.257	0.667
cfPWV	Model 2	Vitamin D, μg/day	-0.028 (0.031)	-0.089 to 0.033	.366	0.667
baPWV	Model 1	Vitamin A, per 100 μg RE/	0.004 (0.029)	-0.053 to 0.061	.897	0.950
baPWV	Model 1	day Vitamin B1, mg/day	-0.284 (0.190)	-0.656 to 0.087	.135	0.667
baPWV	Model 1	Vitamin B2, mg/day	-0.200 (0.178)	-0.548 to 0.148	.261	0.667
baPWV	Model 1	Vitamin B3, mg/day	-0.010 (0.012)	-0.034 to 0.014	.407	0.667
baPWV	Model 1	Vitamin B6, mg/day	-0.138 (0.122)	-0.376 to 0.100	.257	0.667
baPWV	Model 1	Vitamin B9, μg/day	-0.001 (0.001)	-0.003 to 0.002	.579	0.744
baPWV	Model 1	Vitamin B12, μg/day	-0.002 (0.013)	-0.028 to 0.024	.879	0.932
baPWV	Model 1	Vitamin C, mg/day	-0.000 (0.002)	-0.004 to 0.003	.846	0.932
baPWV	Model 1	Vitamin D, μg/day	-0.006 (0.032)	-0.069 to 0.058	.863	0.932
baPWV	Model 2	Vitamin A, per 100 μg RE/	0.001 (0.027)	-0.052 to 0.053	.976	0.976
baPWV	Model 2	day Vitamin B1, mg/day	-0.170 (0.178)	-0.519 to 0.180	.342	0.667
baPWV	Model 2	Vitamin B2, mg/day	-0.142 (0.165)	-0.465 to 0.182	.392	0.667
baPWV	Model 2	Vitamin B3, mg/day	-0.009 (0.011)	-0.030 to 0.013	.433	0.674

Outcome	Model	Vitamin	β (robust SE, HC3)	95% CI	unadjusted *p*-value	FDR *q*-Value
baPWV	Model 2	Vitamin B6, mg/day	-0.116 (0.114)	-0.340 to 0.107	.309	0.667
baPWV	Model 2	Vitamin B9, µg/day	-0.001 (0.001)	-0.003 to 0.001	.326	0.667
baPWV	Model 2	Vitamin B12, µg/day	-0.001 (0.012)	-0.025 to 0.023	.963	0.963
baPWV	Model 2	Vitamin C, mg/day	-0.001 (0.002)	-0.004 to 0.002	.561	0.744
baPWV	Model 2	Vitamin D, µg/day	0.011 (0.030)	-0.047 to 0.069	.716	0.889

FDR correction was applied using the Benjamini-Hochberg procedure across the 36 vitamin-outcome-model contrasts. Model 1 was adjusted for age and sex. Model 2 was additionally adjusted for the number of metabolic syndrome components, the SF-36 general health domain, and Mediterranean diet adherence. Each vitamin was entered as a continuous exposure in a separate model. For vitamin A, coefficients are expressed per 100 µg retinol equivalents/day to improve readability. Inference was based on robust HC3 standard errors and FDR-adjusted q-values. Abbreviations: baPWV, brachial-ankle pulse wave velocity; cfPWV, carotid-femoral pulse wave velocity; CI, confidence interval; FDR, false discovery rate; RE, retinol equivalents; SE, standard error; SF-36, Short Form-36 Health Survey.

Table S4. Diagnostic summary of the primary multivariable models.

Outcome	Model	R^2	Adj.R^2	RSE	SW *p*	BP *p*	Max VIF	Max [rstudent]	Max leverage	Max Cook's D
cfPWV	Model 1	0.285–0.300	0.278–0.293	2.169–2.192	<0.001	<0.001	1.034–1.050	4.420–4.490	0.074–0.310	0.126–0.149
cfPWV	Model 2	0.340–0.351	0.326–0.336	2.119–2.135	<0.001	<0.001	1.207–1.210	4.740–4.840	0.103–0.321	0.191–0.193
baPWV	Model 1	0.406–0.410	0.399–0.404	1.857–1.864	<0.001	0.006	1.034–1.050	5.290–5.400	0.075–0.312	0.162–0.428
baPWV	Model 2	0.472–0.474	0.461–0.463	1.772–1.775	<0.001	<0.0001	1.207–1.210	5.320–5.400	0.103–0.323	0.172–0.252

Values are presented as ranges across the nine vitamin-specific models within each outcome and adjustment set. For BP p in baPWV Model 1, the displayed value corresponds to the midpoint of the observed rounded range across the nine vitamin-specific models. The analytical sample sizes were n = 289 for cfPWV Model 1, n = 283 for cfPWV Model 2, n = 287 for baPWV Model 1, and n = 281 for baPWV Model 2. Model diagnostics included inspection of residuals and fitted values, quantile-quantile plots, variance inflation factor, leverage, studentized residuals, and Cook's distance. Model 1 was adjusted for age and sex. Model 2 was additionally adjusted for the number of metabolic syndrome components, the SF-36 general health domain, and Mediterranean diet adherence. Robust HC3 standard errors were used in the primary regression models. Abbreviations: baPWV, brachial-ankle pulse wave velocity; BP, Breusch-Pagan; cfPWV, carotid-femoral pulse wave velocity; R^2, coefficient of determination; RSE, residual standard error; SF-36, Short Form-36 Health Survey; SW, Shapiro-Wilk; VIF, variance inflation factor..

Table S5. Exploratory effect-modification analyses for the vitamin B1/B6-cfPWV estimates.

Outcome	Vitamin	Effect modifier	Interaction term	β	Robust SE	95% CI	p-value	FDR q-value
cfPWV	Vitamin B1	Sex	Vitamin B1 × sex	-0.147	0.620	-1.362 to 1.068	0.812	0.965
cfPWV	Vitamin B1	Metabolic syndrome burden	Vitamin B1 × SMFR	-0.073	0.191	-0.447 to 0.300	0.701	0.965
cfPWV	Vitamin B1	Mediterranean diet adherence	Vitamin B1 × MD adherence	0.095	0.087	-0.076 to 0.265	0.279	0.965
cfPWV	Vitamin B6	Sex	Vitamin B6 × sex	0.335	0.450	-0.546 to 1.217	0.456	0.965
cfPWV	Vitamin B6	Metabolic syndrome burden	Vitamin B6 × SMFR	0.009	0.121	-0.228 to 0.245	0.943	0.965
cfPWV	Vitamin B6	Mediterranean diet adherence	Vitamin B6 × MD adherence	-0.003	0.062	-0.125 to 0.119	0.965	0.965

Values are unstandardized coefficients for the interaction terms, with robust HC3 standard errors and 95% confidence intervals. All interaction models were estimated using 283 complete cases. Interaction analyses were conducted by adding product terms between vitamin intake and selected effect modifiers to the fully adjusted cfPWV model. The fully adjusted model included age, sex, number of metabolic syndrome components, the SF-36 general health domain, and Mediterranean diet adherence. FDR q-values were calculated across the six exploratory interaction tests using the Benjamini-Hochberg procedure. These analyses were exploratory and were not used to redefine the primary FDR-corrected inference. Abbreviations: cfPWV, carotid-femoral pulse wave velocity; CI, confidence interval; FDR, false discovery rate; MD, Mediterranean diet; SE, standard error; SF-36, Short Form-36 Health Survey; SMFR, number of metabolic syndrome components.

Table S6. Exploratory adjustment of B1/B6–arterial stiffness models for vaccination and SARS-CoV-2 serological markers.

Outcome	Exposure	Model specification	*n*	β	95% CI	p-value	FDR q-value
cfPWV	Vitamin B1	Model 2	283	-0.491	-0.925 to -0.057	0.027	0.109
cfPWV	Vitamin B1	Model 2 + vaccine doses	283	-0.515	-0.968 to -0.062	0.026	0.109
cfPWV	Vitamin B1	Model 2 + SARS-CoV-2 serology	277	-0.484	-0.935 to -0.034	0.035	0.109
cfPWV	Vitamin B1	Model 2 + vaccine doses + SARS-CoV-2	277	-0.508	-0.975 to -0.042	0.033	0.109
cfPWV	Vitamin B6	serology Model 2	283	-0.302	-0.587 to -0.017	0.038	0.109
cfPWV	Vitamin B6	Model 2 + vaccine doses	283	-0.310	-0.601 to -0.018	0.037	0.109
cfPWV	Vitamin B6	Model 2 + SARS-CoV-2 serology	277	-0.273	-0.570 to 0.025	0.073	0.147
cfPWV	Vitamin B6	Model 2 + vaccine doses + SARS-CoV-2	277	-0.275	-0.577 to 0.027	0.074	0.147
baPWV	Vitamin B1	serology Model 2	281	-0.170	-0.520 to 0.181	0.342	0.526
baPWV	Vitamin B1	Model 2 + vaccine doses + SARS-CoV-2	275	-0.127	-0.484 to 0.229	0.483	0.568
baPWV	Vitamin B6	serology Model 2	281	-0.116	-0.341 to 0.108	0.309	0.526
baPWV	Vitamin B6	Model 2 + vaccine doses + SARS-CoV-2 serology	275	-0.053	-0.281 to 0.175	0.645	0.645

Values are unstandardized regression coefficients with 95% confidence intervals, unadjusted p-values, and FDR-adjusted q-values. Model 2 was adjusted for age, sex, number of metabolic syndrome components, SF-36 general health domain, and Mediterranean diet adherence. Vaccine doses refers to the number of COVID-19 vaccine doses received. SARS-CoV-2 serology included N antigen and anti-S IgG levels measured by ELISA. FDR q-values were computed across the exploratory sensitivity models shown in this table. These analyses were exploratory and were not part of the prespecified primary contrast family.

Abbreviations: baPWV, brachial-ankle pulse wave velocity; cfPWV, carotid-femoral pulse wave velocity; CI, confidence interval; ELISA, enzyme-linked immunosorbent assay; FDR, false discovery rate; IgG, immunoglobulin G; SF-36, Short Form-36 Health Survey.

Figure S1. Standardized associations between vitamin intake and central and peripheral arterial stiffness.

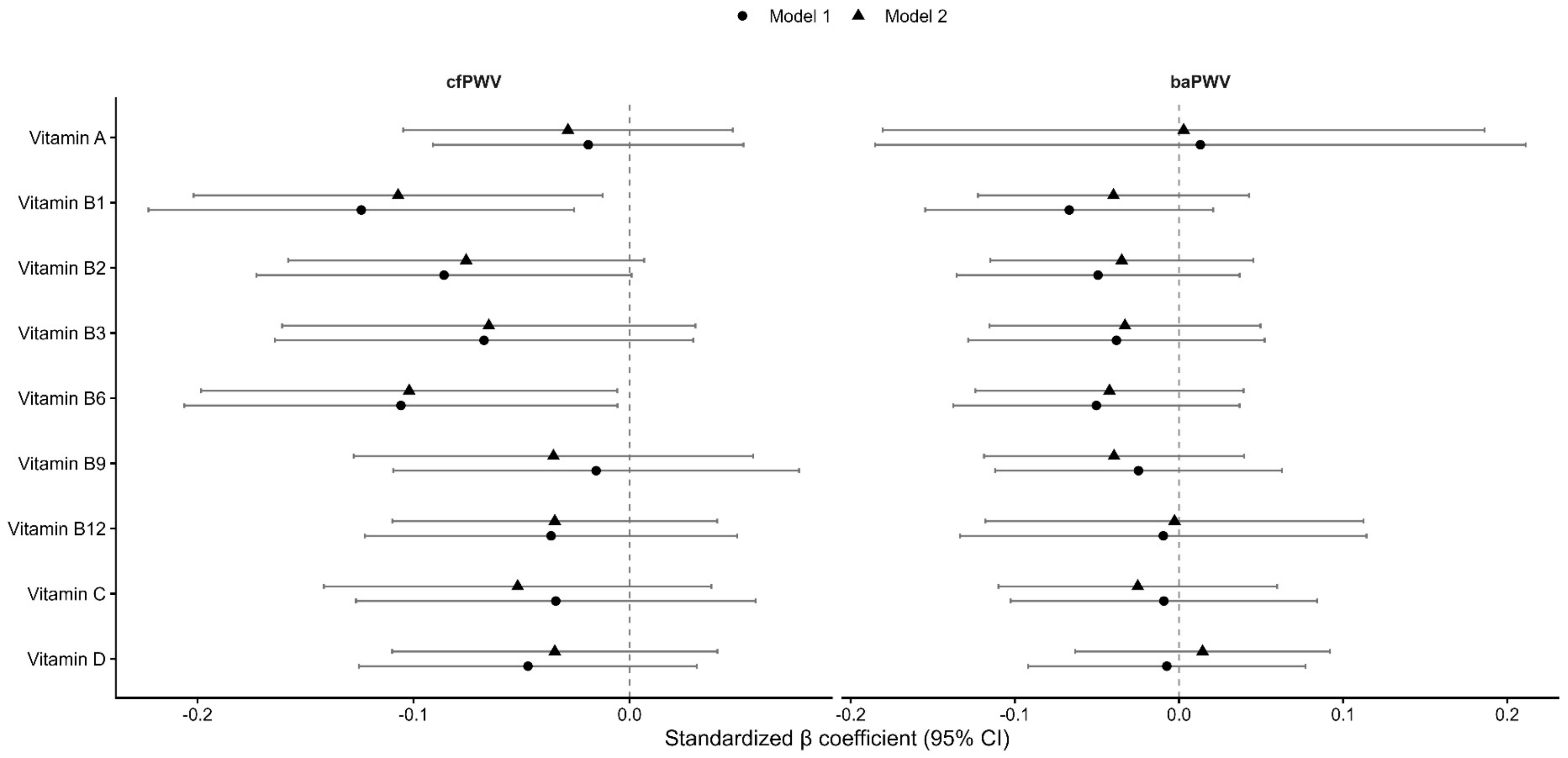

Standardized regression coefficients and 95% confidence intervals are shown for the associations between vitamin intake and cfPWV and baPWV. Standardized coefficients express the change in arterial stiffness, in standard deviation units, associated with a one-standard-deviation increase in vitamin intake, thereby allowing comparison across vitamins measured in different units. Model 1 was adjusted for age and sex. Model 2 was additionally adjusted for the number of metabolic syndrome components, the SF-36 general health domain, and Mediterranean diet adherence. Each vitamin was entered as a continuous exposure in a separate model, and robust HC3 standard errors were used. The vertical dashed line indicates $\beta = 0$. The figure displays the direction and magnitude of

standardized estimates. Statistical significance for the vitamin-arterial stiffness association analyses was determined using FDR-adjusted q-values; no association remained statistically significant after FDR correction. Abbreviations: baPWV, brachial-ankle pulse wave velocity; cfPWV, carotid-femoral pulse wave velocity; CI, confidence interval; SF-36, Short Form-36 Health Survey.
